# Aboveground Tree Growth Varies with Belowground Carbon Allocation in a Tropical Rainforest Environment

**DOI:** 10.1371/journal.pone.0100275

**Published:** 2014-06-19

**Authors:** James W. Raich, Deborah A. Clark, Luitgard Schwendenmann, Tana E. Wood

**Affiliations:** 1 Department of Ecology, Evolution & Organismal Biology, Iowa State University, Ames, Iowa, United States of America; 2 Department of Biology, University of Missouri-St. Louis, St. Louis, Missouri, United States of America; 3 School of Environment, The University of Auckland, Auckland, New Zealand; 4 United States Department of Agriculture Forest Service, International Institute of Tropical Forestry, Rio Piedras, Puerto Rico; 5 Fundación Puertorriqueña de Conservación, San Juan, Puerto Rico; Tennessee State University, United States of America

## Abstract

Young secondary forests and plantations in the moist tropics often have rapid rates of biomass accumulation and thus sequester large amounts of carbon. Here, we compare results from mature forest and nearby 15–20 year old tree plantations in lowland Costa Rica to evaluate differences in allocation of carbon to aboveground production and root systems. We found that the tree plantations, which had fully developed, closed canopies, allocated more carbon belowground - to their root systems - than did mature forest. This increase in belowground carbon allocation correlated significantly with aboveground tree growth but not with canopy production (*i.e*., leaf fall or fine litter production). In contrast, there were no correlations between canopy production and either tree growth or belowground carbon allocation. Enhanced allocation of carbon to root systems can enhance plant nutrient uptake, providing nutrients beyond those required for the production of short-lived tissues such as leaves and fine roots, and thus enabling biomass accumulation. Our analyses support this deduction at our site, showing that enhanced allocation of carbon to root systems can be an important mechanism promoting biomass accumulation during forest growth in the moist tropics. Identifying factors that control when, where and for how long this occurs would help us to improve models of forest growth and nutrient cycling, and to ascertain the role that young forests play in mitigating increased atmospheric carbon dioxide.

## Introduction

Forests provide a variety of products and services to human societies, sustain diverse flora and fauna and, through their interception and processing of solar energy and precipitation, influence climate and the composition of the atmosphere [1], [2]. Continued loss of forest cover, and accelerating rates of loss in the tropics [3], impart importance to the understanding of processes that promote forest growth and of recovery of the benefits they provide [4]. For example, biomass accrual in secondary forests and plantations partially mitigates the carbon dioxide (CO_2_) emissions that result from land use changes [5]. Biomass accumulation during forest stand development is one of the most paradigmatic processes in ecology [6], [7] and, on one level, is readily understood: the growth of a cohort of small trees to larger sizes through time reflects biomass accrual at the stand level. However, the processes that control the strength and duration of carbon (C) accumulation in growing forests remain poorly defined. Rates of biomass C accumulation can be particularly rapid in young tropical forests and plantations [8], [9], [10], but sustaining rapid tree growth requires substantial supplies of nutrients [11]. This implies, and we propose, that rates of aboveground biomass accumulation and belowground C allocation (BCA) in growing tropical forests are coupled, such that both processes vary together and independently of canopy production. We tested these hypotheses, and alternatives, using evidence provided by extensive measurements of key aspects of C cycling in young plantations and mature forest stands growing on the same soils in lowland Costa Rica.

Forest growth depends upon the capture of aerial resources, primarily sunlight and CO_2_, by leaves, and of soil resources, primarily water and nutrients, by fine-root systems (we use this terminology to emphasize that mycorrhizas are specifically included). Increased rates of leaf production and tree growth must be balanced by increased uptake of soil resources by root systems. Plants may adjust their allocation of available resources to balance the uptake of multiple limiting resources [12], [13]. However, there is a fundamental difference between allocation of assimilated C to leaves versus to fine-root systems. Leaves absorb, reflect and transmit incoming solar radiation and, in so doing, reduce the amount of light reaching lower leaves. Once full canopy coverage has developed in a growing forest stand, the production of additional leaf area provides a diminishing return of photosynthetic products [14]. Photosynthesis saturates at high leaf area index (LAI, the area of leaves per unit ground area), although other factors such as leaf nitrogen (N) content and display angle are also involved [15]. Maximum LAI may develop relatively quickly in growing forests but does not thereafter increase, and may decrease [16].

Does a similar limit exist belowground? It is theoretically possible. Fine roots and mycorrhizas absorb nutrients from the soil and deplete soil nutrient concentrations within the rhizosphere, with the extent of depletion diminishing with distance from an absorbing root or hyphal surface [17]. At high densities, fine-root systems could efficiently capture available soil nutrients such that growth and maintenance of more extensive root systems would have diminishing values. However, in contrast to many theoretical and empirical studies that support the existence of a maximum sustainable leaf area, there is no parallel evidence that forest soils frequently are saturated with fine-root systems. Young secondary forests and plantations in the moist tropics can grow very rapidly [9], [10]. However, tree biomass contains nutrients and biomass accumulation entails nutrient sequestration in relatively long-lived woody tissues. Rapidly growing tropical forests may therefore be particularly prone to developing nutrient limitations. Increased allocation of newly fixed C to the support, regeneration, and expansion of fine-root systems can enhance plant nutrient uptake. By increasing the amount of absorbing surface area per volume of soil, more extensive fine-root systems may more effectively intercept available soil nutrients [17]; more effectively exploit nutrient-rich hot spots and transient pulses of nutrient availability [18]; and generally compete more successfully with soil microbes for nutrients. Some tree species produce root nodules that support mutualistic N-fixing bacteria [19]. Almost all trees enhance their nutrient uptake capacities by supporting mycorrhizas [20]. Many plant roots release organic compounds into the soil that promote soil organic matter turnover and nutrient availability to plants [21], [22], [23]. Via these and other processes, greater plant allocation of available substrates to fine-root systems may improve plant nutrition and thereby enhance forest growth. Greater allocation of C belowground could be a common feature of growing forests (*e.g*., [24]).

The word ‘allocation’ has been used variably within plant physiology and forest ecology literatures [25]: we herein use ‘allocation’ to indicate a flux of organic substrates to specific forest components, with units of mass area^−1^ time^−1^. Aboveground, we distinguish canopy production and tree growth, with allocation to canopy production being empirically determined from measurements of fine litterfall including leaves, and aboveground tree growth being equivalent to aboveground biomass increment (ABI). We also use leaf fall, the leaf component of fine litterfall, as a proxy for leaf production. We sum canopy production and tree growth to provide a widely used [26], [27] estimate of aboveground net primary productivity (ANPP). Belowground, we consider only total BCA, which includes both belowground NPP and respiratory CO_2_ from roots and their rhizospheres. We do so because cleanly distinguishing the specific pathways of C flux belowground remains fraught with uncertainty: this does not influence the comparisons we make between specific fluxes measured across sites. We do not use ratios or proportions to express allocation without explicitly stating so, to avoid confusion with ‘partitioning’, which refers to the proportion of total photosynthesis (GPP) that is utilized by a particular forest component or process [25]. We recognize that canopy production implies more than light capture for photosynthesis by leaves: it includes leaves but also twigs (≤1 cm diameter), meristems, and flowers and fruits. In addition to being the site of photosynthesis, plant canopies perform nitrate and sulfate reduction, and supply nutrients to roots [28]. They also produce and sense plant growth hormones that affect C allocation to roots and shoots [29].

Analysis of compiled data from a range of primarily mature and moist tropical forests indicated that NPP was distributed among canopy, wood, and fine root components in mean proportions of 34, 39 and 27%, respectively, and that variations from these mean proportions reflected a trade-off between fine roots and wood [27]. A similar conclusion was derived with a data-tested model of C allocation strategies of trees in old-growth forests [30]. Our proposition of a positive relationship between BCA and tree growth in young tropical forests contradicts these ideas. Nevertheless, based on the theoretical considerations described above, we suggest that growing forests may necessarily be more flexible in their allocation of production, to successfully balance nutrient uptake with aboveground growth [10], [11], [18].

**Figure 1:**
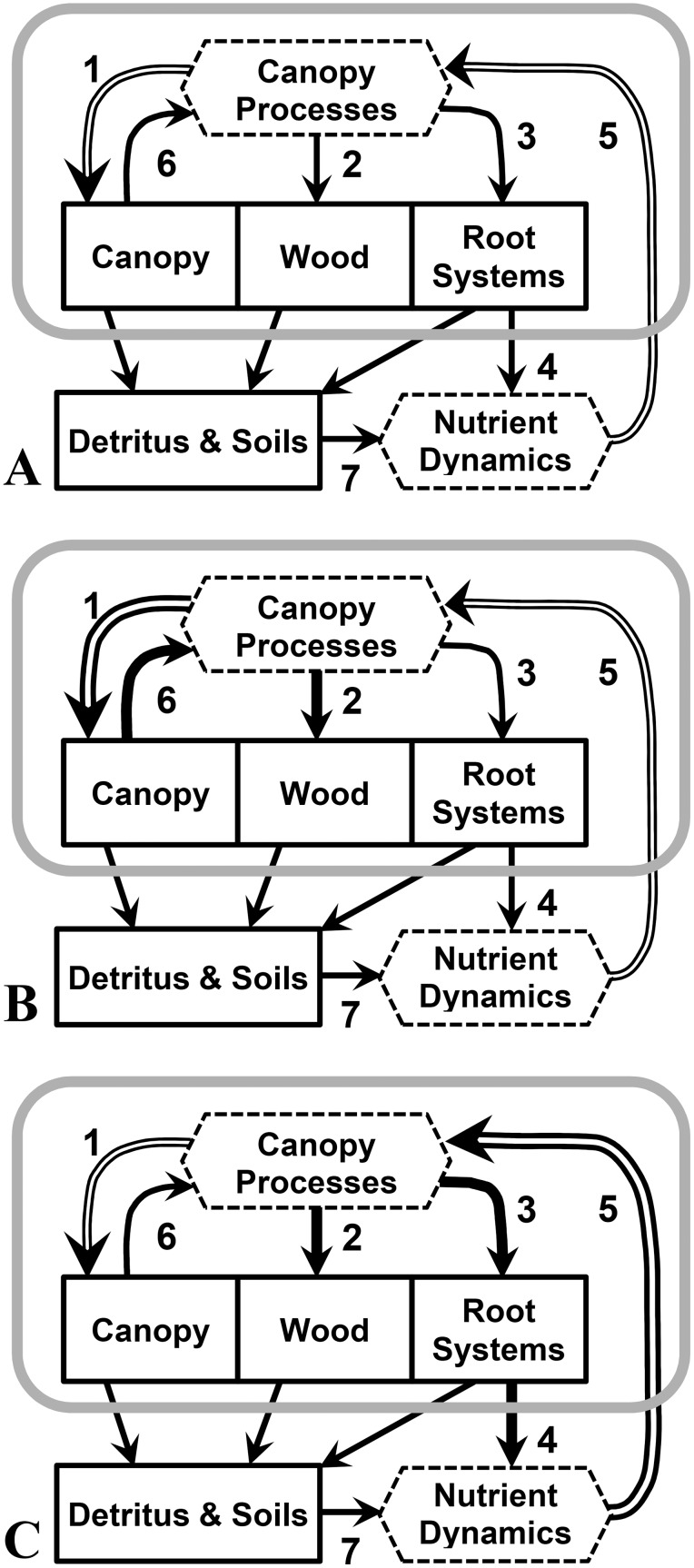
Schematic representation of alternative scenarios of organic matter flows through forest vegetation. Plant processes are encircled in gray, below which are soil processes: CO_2_ uptake (photosynthesis) and release (respiration) are not depicted. Solid lines are organic matter flows; the two hollow arrows represents represent fluxes of organic matter and nutrients; solid boxes represent organic matter stocks (mass area^−1^); and the two hexagons represent material transformations. Numbered flows represent: 1, allocation of photosynthates to the canopy; 2, allocation of NPP to woody tissues; 3, belowground carbon allocation (BCA); 4, root-system effects on soil nutrient availability; 5, root-system fluxes to canopy processes, *e.g*., nutrient uptake; 6, feedbacks from canopy processes to NPP; and 7, transformations of detrital and soil nutrients into plant-available forms. Additional arrows show fluxes of plant detritus to soils; and transformation of detrital and soil nutrients into plant-available forms (flow 7). Three alternative scenarios are shown, with thicker arrows representing relatively larger fluxes. **A)** Fixed-allocation scenario, with organic matter fluxes to the canopy (flow 1), to wood production (flow 2), and to root systems (flow 3) varying uniformly. **B)** Canopy-feedback scenario, with relatively greater allocation of photoassimilates to the canopy (flow 1) and back (flow 6) providing a positive feedback to ANPP. **C)** Belowground-feedback scenario, with increased BCA stimulating nutrient uptake (flow 5) and wood NPP (flow 2).

We used a case-study approach to test alternative scenarios of forest C allocation (Fig. 1), which served as multiple working hypotheses *sensu* [31], by directly comparing data from concurrent studies of mature forest and replicated 15–20 year old plantations of native tree species located in close proximity to one another on the same highly weathered soils in lowland Costa Rica. Our primary objective was to assess whether between-site differences in tree growth rates (*i.e*., wood productivity) could be ascribed to (I) differences in canopy production (scenario B in Fig. 1), (II) differences in BCA (scenario C in Fig. 1), or (III) proportionally equivalent differences in both processes (scenario A in Fig. 1). As illustrated in Figure 1, allocation of substrates to canopy production (flow 1) can generate a positive feedback to NPP (flow 6). In that case (Fig. 1B) a positive correlation between tree growth and canopy production is expected (*e.g*., [26]). Similarly, preferential allocation of available substrates to root systems (BCA, flow 3 in Fig. 1) may stimulate soil nutrient turnover and uptake (flow 4), also producing a positive feedback to NPP (flow 5), in which case (Fig. 1C) tree growth and BCA should correlate positively. If available substrates are allocated in consistent proportions to leaves, to wood, and to root systems (Fig. 1A), *i.e*., with ‘fixed allocation coefficients’ [27], [32], then canopy production, tree growth, and BCA will all correlate with one another across both growing and mature forest plots.

To fulfill our objective we tested multiple hypotheses that together made it possible to distinguish among the alternative allocation scenarios depicted in Figure 1. First, to assess possible differences in canopy attributes related to productivity (‘canopy processes’ in Fig. 1), we hypothesized that (H1a) total leaf area, (H1b) leaf litter production (*i.e.*, leaf fall), (H1c) N concentrations in leaf fall, and (H1d) leaf fall N fluxes were similar between the plantations and mature forest. Second, to determine if the two sites differed in aboveground productivity, we tested the hypotheses that (H2a) litterfall, (H2b) aboveground tree growth, and their sum, (H2c) ANPP, were similar. To identify belowground differences, we tested the hypothesis (H3) that BCA (flow 3 in Fig. 1) was greater in the plantations than in the mature forest. Belowground C allocation is an empirically based assay of the amount of photosynthetically produced substrate that is transported from the canopy through the phloem to root systems in forests [33]. Lastly, to determine which of the three alternative allocation scenarios (Fig. 1A, B, C) best describes observed differences among mature forest and plantation plots, we applied correlation analyses.

## Methods

### Ethics Statement

All field studies were conducted on land owned by the Organization for Tropical Studies, who gave permission for this research. The research also was approved and permitted by the Costa Rican Ministerio de Ambiente y Energía. We confirm that the field studies did not involve sampling of any endangered or protected species.

### Study Area

Field studies were conducted at La Selva Biological Station of the Organization for Tropical Studies, in northeastern Costa Rica (10°26′N, 83°59′W). Annual rainfall from 1997–2009 averaged 4537 mm, air temperatures averaged 25.1°C, and elevations of the study sites ranged from 37–150 m [10], [34]. The driest season at La Selva normally extends from February through April, but every month averages >150 mm of rain. The vegetation of the mature forest was species-rich broad-leaved lowland evergreen forest with abundant palms and a relatively high abundance of the canopy tree *Pentaclethra macroloba* (Fabaceae). The forest contained an average of 550 trees ha^−1^ (stems ≥10 m diameter) with a basal area of 24 m^2^ ha^−1^ [35]. The principal mature forest study sites are described in more detail elsewhere [34], [35]; we utilized data from the slope and plateau plots (twelve 0.5-ha plots) in the CARBONO plot network.

The experimental plantations were established on sites that previously were mature forest that was cut, burned and converted to pasture in the 1950’s, and that remained in low-maintenance pastureland until the cattle were removed and the trees were planted, from June 1988 through February 1989. The 50-m×50-m plantation plots were established as a complete randomized block experiment having four blocks and one tree species per plot, and now have diverse and luxuriant understories [10]. The blocks originally contained 12 plots each, 11 planted to individual tree species and one left as an unplanted control. Each block draped over a single hill and thus included all hilltop and slope topographies. Seedlings were planted at 3-m×3-m spacing and plots of the fastest growing species were thinned at age four [36], [37]. We utilize data from 16 plantation plots, four replicates each of four locally native tree species: *Hieronyma alchorneoides* Allemäo (Phyllanthaceae), *Pentaclethra macroloba* (Willd.) Kuntze (Fabaceae), *Virola koschnyi* Warb. (Myristicaceae), and *Vochysia guatemalensis* Donn. Sm. (Vochysiaceae). Plantation understories were manually cleared of competing vegetation over the first four years [37] and saplings of *Vochysia guatemalensis*, which were seeding into other plots, were cut out in early 2006. To minimize edge effects the outer two rows of trees were not sampled; measurements were restricted to the central 0.13-ha subplot that originally contained 12 rows of 12 trees each. During this study, stem density in the plantations averaged 314 trees ha^−1^, basal area averaged 18 m^2^ ha^−1^, and the planted trees were 15–21 years old.

Both the mature forest and plantation plots were located on the same soil type, a Typic Tropohumult according to [38], a Haplic Haploperox according to [39]. This soil is commonly referred to as residual soil in literature from La Selva, and we use that descriptor hereafter. The residual soils are deep, highly weathered, well-drained clays derived from very old basaltic lava flows. They are acidic, have high amounts of exchangeable acidity and low base saturation, are relatively high in organic matter, and are free of stones and gravel in the surface 3 m [38], [39]. The plantation experiment was designed to investigate the capacities of different tree species to improve soil conditions in degraded pasturelands [40]. Direct comparisons of soils from different plantation tree species at age 15 years [41] documented significant tree-species effects on soil properties, and demonstrated lower soil N contents in the plantations than in an adjacent mature forest site on residual soil. No differences in Olsen-extractable P in surface soils were observed between mature forest and plantation soils [41]. Based on those data from the plantation plots [41] and comparable measurements of soils from the mature forest plots [42], there were no significant differences between the mature forest and plantations in surface soil pH (range 4.0–4.8 in water) or in SOC stocks to 30 cm depth (range 83–90 Mg ha^−1^) (Table S1). However, there was significantly less soil N (95% CI 6.6–7.03 versus 7.05–8.3 Mg ha^−1^) and higher soil C: N (12.4–12.9 versus 11.8–12.2) in the plantations than in the mature forest (0–30 cm depth, Table S1). Soil P stocks may also differ between the sites, but the different analytical techniques used in the two studies preclude direct comparison.

### Field and Analytical Methods

To test our hypotheses, we integrated previously published and unpublished data from our field studies of the mature forest and plantations (Table S1). Most comparisons were based on concurrent studies conducted during 2003–2009, although exact time-matching was not always possible. Comparable methods were used in the two sites in most cases, as described below. Further methodological details are provided in Table S1 and in the source publications cited therein.

The aboveground biomass of each tree ≥10 cm diameter in each mature forest plot was estimated annually from tree diameter measurements, using the tropical wet forest regression of [43]. In the plantations, the diameters (*d*) and heights (*h*) of each tree in each plot were measured annually, and total aboveground biomass of each tree was estimated from *d* ^2^ *h* based on species-specific regressions that were derived from the harvest of nine trees of each of the planted species from the experimental plots (Table S2). The harvested trees encompassed the entire size range of each species, including the largest individuals. An average of 8% of the tree biomass in the plantations was comprised of native tree species that naturally colonized the plots; the aboveground biomass of each of those trees (≥10 cm diameter) was similarly estimated with allometric equations derived from harvests of 49 trees from seven species (Table S2).

Annual aboveground tree growth was determined for each surviving tree each year as the difference in estimated biomass between years. The net annual change in aboveground biomass in each plot of each site was determined by difference between the plot-level biomass estimates derived from the 2003 and 2009 inventories. Aboveground fine-litter production (*i.e.*, litterfall) was measured in each plot with litter traps that were emptied at *ca.* two-week intervals from 2003–2009 (Table S1). Litterfall excluded branches >1 cm diameter and, in the mature forest, frass. We estimated aboveground NPP in each plot by summing tree growth and litterfall. Litterfall was sorted into components and the N flux in leaf fall was determined by multiplying its mass by its N content. Mature forest leaf fall samples from three collection dates per year (4–5 month intervals over 1997–2001, [44]) from all 12 forest plots were analyzed using a modified Kjeldahl digestion followed by colorimetric analysis on an Alpkem Flow Solution IV Autoanalyzer (OI Analytical, College Station, Texas, USA). Monthly leaf fall samples from each of the 16 plantation plots were analyzed with an elemental analyzer (Flash EA1112 CN Analyzer, CE Elantech, Lakewood, New Jersey, USA) over three years (2004, 2005, 2009).

Leaf area index was measured differently in the mature forest and plantations. In the mature forest, all leaves within 45 randomly located 1-m^2^ vertical transects within old-growth forest were harvested and measured via repeated assembly and disassembly of a scaffolding tower [45]. In the plantations, the leaf areas of each of the planted trees, of all other trees ≥10 cm in diameter, and of saplings 2.5–10 cm in diameter were determined allometrically, based on regressions of total-tree leaf area from stem diameter (Table S2), with total-tree leaf area being determined from leaf biomass multiplied by the specific leaf mass (g m^−2^) of each harvested tree. Leaf area of understory vegetation, including all non-woody plants and woody plants with diameters <2.5 cm diameter, was measured by direct harvests of four 0.5-m^2^ quadrats per plot in 2005 [10]. Annual measurements of the diameters of trees and saplings by species were used to determine their total plot-level leaf area by year, and these were added to the 2005 understory leaf area (average 0.7 m^2 ^m^−2^) to quantify total leaf area of each plot [10].

Total CO_2_ emissions from the soil surface, *i.e.*, soil respiration rates (R_SOIL_), were determined at approximately monthly intervals using dynamic closed chamber systems attached to LI-COR gas analyzers [10], [46]. In the mature forest, eight aluminum chambers (0.2 m in diameter, 0.15 m tall) were installed to about 0.02 m into the soil along four parallel transects in each of three 0.5-ha plots. Once inserted, the chambers were left in place and kept free of vegetation throughout the whole study period. Air was circulated at a flow rate of about 0.8 L min^−1^ between an infrared CO_2_ gas analyzer (LI-800, Li-Cor Inc., Lincoln, NE, USA) and the flux chambers for about 5 min. To prevent pressure differences between chamber and atmosphere, chambers were vented to the atmosphere through a 0.025-m long stainless-steel tube. CO_2_ concentrations were recorded at 5 s intervals with a datalogger (CR800, Campbell Scientific, Logan, UT, USA). Soil respiration rate was calculated from the linear change in CO_2_ concentration multiplied by the density of air and the ratio of chamber volume to soil surface area. The infrared gas analyzer was calibrated in the lab using nitrogen as zero standard and a CO_2_ standard (450 ppm). Measurements were conducted between 8 AM and 2 PM local time. For each of the sites the average soil respiration rate was calculated from the eight chamber flux measurements on a sampling day. Daily mean soil respiration for each site was calculated by linear interpolation between sampling dates. Daily CO_2_ flux rates were then cumulated to estimate annual flux rates. In the plantations, soil respiration over 2004 through 2005 was measured in 3–4 locations per plot with an LI-8100 automated soil CO_2_ flux system and 8100-102 (0.10-m diameter) chamber (LI-COR Biosciences, Lincoln, Nebraska, USA) [10], [47]. From 2007–2010 soil respiration was measured over two full years at three-week intervals in four locations per plot using the same system attached to an 8100-103 (0.20-m diameter) chamber. Mean fluxes were not affected by this change, but within-plot variability was reduced. Soil collars were 0.12 m tall and were inserted about 0.02 m into the surface soil. Living plants were removed from inside the collars, but surface litter and branches <5 cm diameter were left. Within-chamber CO_2_ concentrations were measured every second for 3–5 minutes. The resulting CO_2_-concentration time series were analyzed using FV8100 software from LI-COR. Collar locations were moved annually or whenever they were disturbed. The LI-8100 was factory calibrated annually. Data from the plantations were averaged within months, and those monthly means were summed to estimate the mean annual flux, because sampling there did not include all months every year: in the mature forest each month was sampled each year. Belowground C allocation was estimated as R_SOIL_ minus litterfall-C. This approach is likely to underestimate total BCA because it excludes woody-root biomass accumulation, which is particularly important in growing forests [48]. For C budgeting, biomass components in the mature forest were assumed to be 48% C, similar to those in the plantations.

Belowground C allocation (flow 3 in Fig. 1) is a focus of our study (Fig. 1a) and so deserves further explication. The approach taken to estimate BCA included only the major belowground C fluxes that were directly measured in both sites, thus allowing for a robust comparison. As in the case of ANPP, however, several minor fluxes were not measured for this study. Measurements of some of these other minor fluxes have been made at La Selva, in forests on residual soils (Table S3). The mass of fine litter on the soil surface (*i.e*., the forest floor) was similar in the mature forest and the plantations [49], so net accumulations of forest floor C were negligible. Net increments of soil organic C (SOC) stocks averaged 0.23 Mg ha^−1^ year^−1^ in the plantations [10] but were miniscule in the mature forest [50]; we also assumed those to be negligible in both sites. In the mature forest, inputs of dissolved organic and inorganic C to the soil in canopy throughfall were small, and were balanced by equivalent (within measurement error) leaching losses (Table S3); a similar situation was presumed to exist in the plantations. We have no estimates of dissolved CO_2_ fluxes in xylem sap water [51], but similarities in canopy characteristics between the forests being compared, which were at the same location on similar soils and landscape positions, minimize the likelihood of there being quantitatively important differences among the sites. It is likely that soils in both sites consumed some atmospheric methane, but at a very low rate [52]. Ignoring these minor and unmeasured soil C fluxes does not bias the comparison we make between BCA in the mature forest and plantations. We did not include estimates of net belowground coarse root accumulation within our estimates of BCA because coarse roots (including belowground stumps and stems) have not been measured in both sites, but we provide estimates of the possible magnitude of that exclusion (Table S3) based on root-shoot biomass ratios derived from other sites [53]. Inclusion of that flux would likely increase the difference in BCA between the mature forest and plantations, by perhaps 0.5 Mg ha^−1^ year^−1^ (Table S3).

### Statistical Analyses

To compare data from mature forest plots to those from plantation plots, we determined the arithmetic means of the plot-level data and determined their 95% confidence intervals (*CI*) using bootstrapping. Specifically, for each variable of interest within each site we used resampling with replacement to generate 5000 equal-sized populations of plots, confirmed that their average mean matched the empirically determined value, and then excluded the smallest and largest 2.5% to characterize the *CI*. In the plantations, bootstrapped populations were equally distributed among the four planted tree species. Bootstrapping is particularly appropriate for estimating confidence intervals when some sample sizes are low and heterogeneity among variances may exist [54]. For most variables sample sizes were 12 mature forest plots and 16 plantation plots (Table S1). To distinguish among the alternative scenarios of allocation of production (Fig. 1) we performed correlation analyses using the Restricted Maximum Likelihood (REML) method of JMP (Version 10.0.0, SAS Institute, Inc.). Bootstrapping was performed using the data analysis toolpack of Microsoft Excel 2010.

## Results

**Figure 2:**
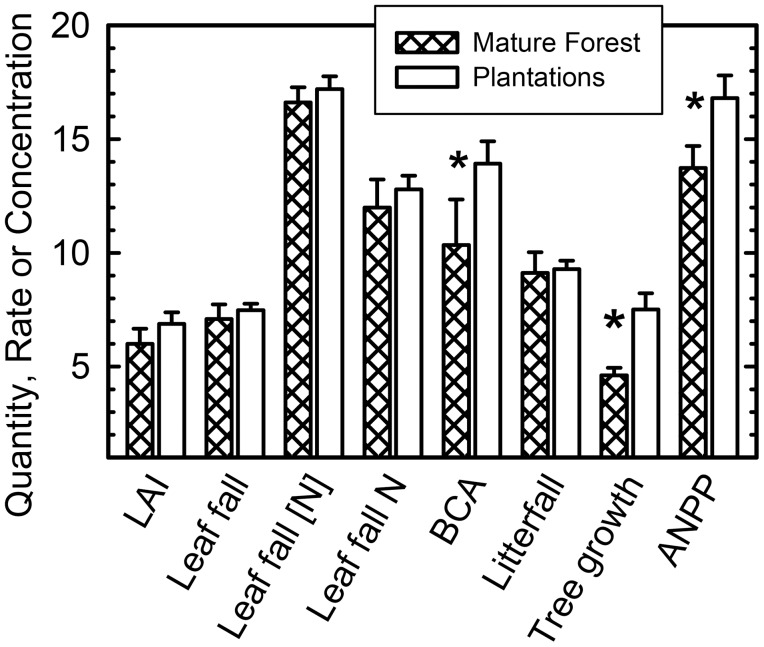
Comparisons of attributes of mature forests and plantations of native tree species at La Selva, Costa Rica. Variables are, left to right: total leaf area index (LAI, m^2 ^m^−2^); annual leaf fall (Mg ha^−1^); the mean N content of leaf fall (mg g^−1^); the N flux in leaf fall (10^4 ^g ha^−1^ year^−1^); belowground carbon allocation (BCA, Mg ha^−1^ year^−1^); litterfall (Mg ha^−1^ year^−1^); annual aboveground tree growth (Mg ha^−1^); and aboveground net primary productivity (ANPP, Mg ha^−1^ year^−1^). Statistically significant differences based on bootstrapped confidence intervals are identified with an asterisk (*). Additional information is provided in Table S1.

We identified both similarities and significant differences between plantation and mature forest plots (Fig. 2, Table S1). Leaf fall was similar in the two sites (*P*>0.1), averaging (±1 *SEM*) 7.3±0.2 Mg ha^−1^ year^−1^ (*n* = 28). Leaf fall N contents were measured in different years in the two sites but, based on those data, neither N concentrations in leaf fall (*n* = 28, 17.0±0.4 mg g^−1^) nor leaf litter N fluxes (*n* = 28, 124±4 kg ha^−1^ year^−1^) varied significantly between sites. Total leaf area index over 2003–2005, when the mature forest data were collected, averaged 6.0±0.3 m^2 ^m^−2^ (*n* = 45) in the mature forest and 6.9±0.5 m^2 ^m^−2^ (*n* = 16) in the plantations, but these were not statistically different due to high variability among plots (0.5<*P*<0.10). Aboveground NPP was greater in the experimental plantations (*CI* 15.8–17.8 Mg ha^−1^ year^−1^) than in the mature forest (12.9–14.7 Mg ha^−1^ year^−1^, Fig. 2). This difference was due to greater tree growth in the plantations: total fine litterfall did not differ significantly between the sites (*n* = 28, 9.2±0.3 Mg ha^−1^ year^−1^). Tree growth, in contrast, averaged 60% faster in the plantations (mean 7.5, *CI* 6.8–8.2 Mg ha^−1^ year^−1^) than in the mature forest (mean 4.6, *CI* 4.3–4.9 Mg ha^−1^ year^−1^, Fig. 2). Belowground C allocation also was greater in the plantations (13.0–14.9 Mg ha^−1^ year^−1^, *n* = 16) than in mature forest (8.3–12.3 Mg ha^−1^ year^−1^, *n* = 3), which had less R_SOIL_ (Table S1).

**Figure 3:**
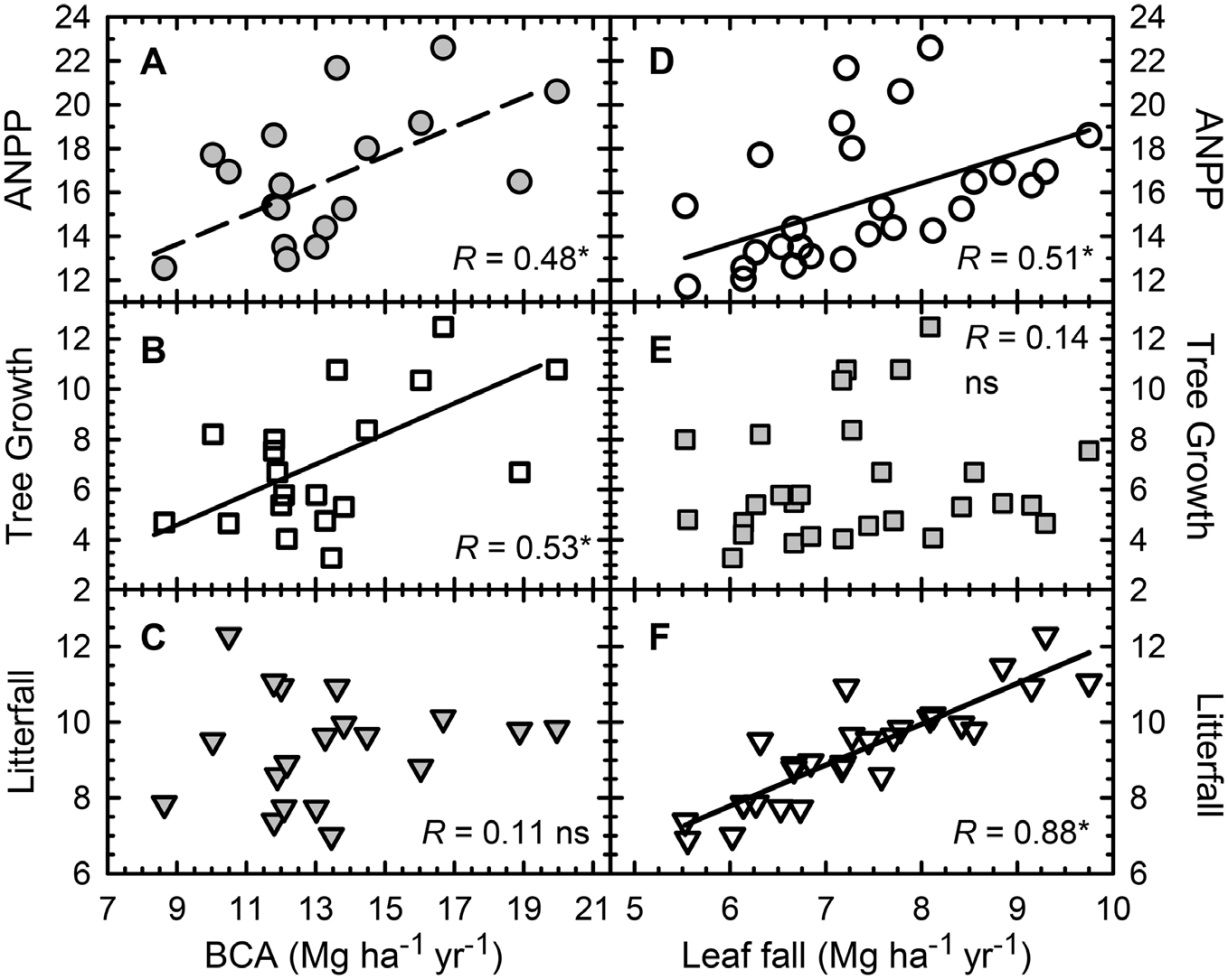
Belowground C allocation correlated positively with ANPP (A) and with tree growth (B) but not with litterfall (C). Leaf fall, in contrast, correlated with ANPP (D) and with litterfall (F), but not with tree growth (E). Lines show linear trends for significant relationships. Values are annual means in Mg ha^−1^ of organic matter, except that BCA is in units of C.

**Figure 4:**
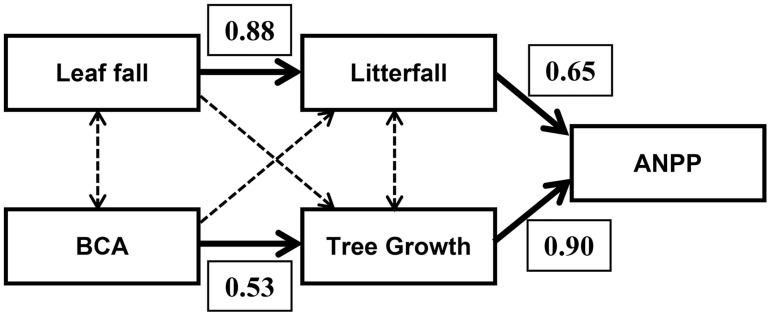
Correlations among carbon-flux variables used to distinguish among the alternative allocation scenarios depicted in **Figure 1**. Dashed arrows indicate insignificant relationships.

Across all plots regardless of stand age, both tree growth and ANPP increased with increasing BCA, but litterfall did not (Fig. 3). Both litterfall and ANPP increased with increasing leaf fall, but tree growth did not (Fig. 3). Correlation analyses (Table S4) indicated two distinct pathways of influence. Leaf fall comprised an average of 79% of fine litterfall and so those two variables were strongly correlated (*r* = 0.88), but leaf fall was not related to either tree growth or BCA (Fig. 4). Belowground C allocation, in contrast, correlated significantly with aboveground tree growth (*r* = 0.53) but not with either leaf fall or litterfall (*P*>0.1).

## Discussion

Rapid growth and biomass accumulation by tropical secondary forests and plantations partially mitigate C losses to the atmosphere that accompany deforestation [5] and promote restoration of ecosystem services on deforested lands [8], [55]. However, rapid forest growth requires nutrients. A case example is provided by rapidly growing plantations of four native tree species in lowland Costa Rica. There, C sequestration rates in biomass averaged >5 Mg ha^−1^ year^−1^ over the first 16 years of tree growth and were still high, nearly 4 Mg C ha^−1^ year^−1^, at age 16 [10]. This rapid C sequestration was accompanied by very rapid rates of N uptake by the vegetation, which averaged 350 kg N ha^−1^ year^−1^, much of which was derived from soil N stocks [56]. It may be difficult to sustain such rapid tree growth and nutrient uptake. Nutrient limitations are widespread [57], [58] and potentially depress tree growth and stand-level biomass accumulation rates [11], [59]. We asked “What processes promote nutrient uptake by, and alleviate nutrient limitation to, these rapidly growing tropical forest stands?” We theorized that additional allocation of C to root systems in growing forests could promote the uptake of soil nutrients needed to support tree growth. We specifically conjectured that rates of aboveground biomass accumulation and belowground C allocation were linked, such that both processes would vary together and independently of leaf or canopy production, other factors being similar.

We tested these ideas by putting forth three alternative scenarios of C allocation (Fig. 1) and testing their predictions based on combining all relevant plot-level data from both sites. In the fixed-allocation scenario (Fig. 1A) BCA, tree growth and canopy production would all correlate with one another across both plantation and mature forest plots. However, neither BCA nor tree growth were significantly related to canopy production, measured as either leaf fall or total fine litter production (Fig. 4). In the canopy-feedback scenario (Fig. 1B) positive correlations between canopy production (flow 1 in Fig. 1) and tree growth (flow 2 in Fig. 1) are predicted. We found that leaf fall and litterfall provided virtually equivalent measures of canopy production (*r* = 0.88), but tree growth was not related to either variable (Fig. 4, Table S4). The belowground-feedback scenario (Fig. 1C), in contrast, predicts a positive correlation between BCA and tree growth, but no correlation between BCA and canopy production. This scenario is consistent with our findings: aboveground biomass increments (*i.e*., tree growth) increased with increasing BCA whereas there was no correlation between BCA and canopy production (*P*>0.4).

**Figure 5:**
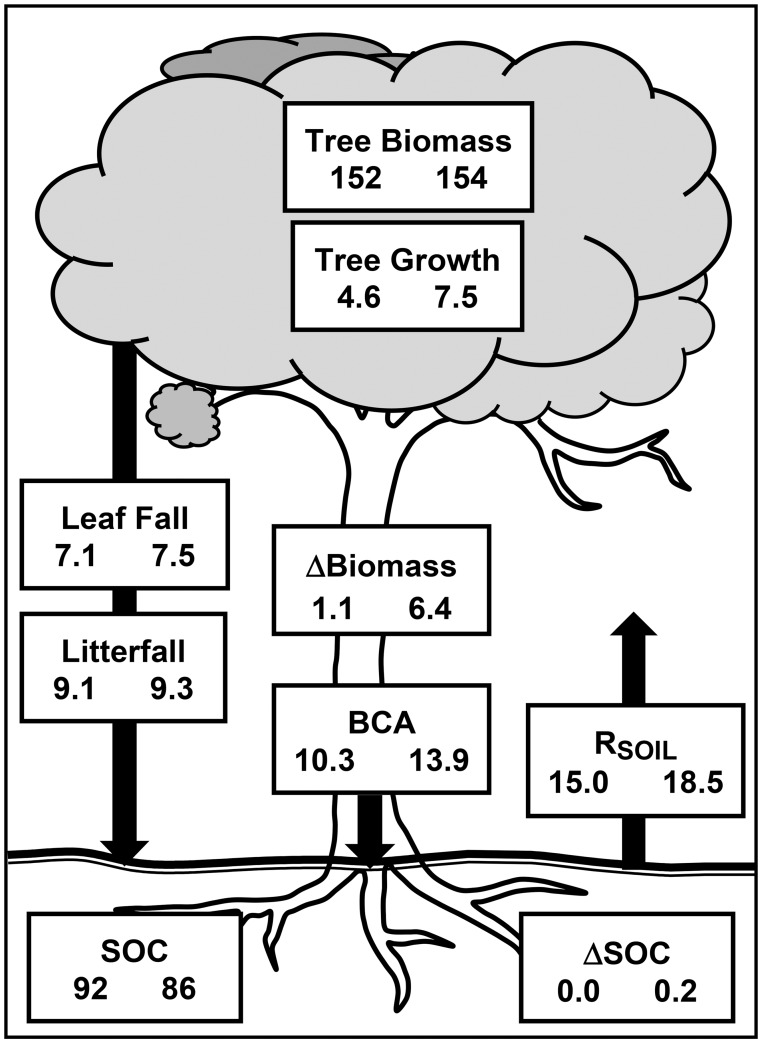
Carbon-cycling characteristics of mature rainforest and nearby tree plantations at La Selva, Costa Rica. In each box the value at the left is the mean annual value for the mature forest, and the value at right is the mean annual value of 15–20 year old plantations. BCA is belowground carbon allocation; R_SOIL_ is soil respiration; and ΔBiomass refers to the net annual increment in total aboveground tree biomass from 2003–2009; root biomass in excluded. Units are Mg ha^−1^ of biomass except for BCA and R_SOIL_, which are in units of carbon. All fluxes are annual; data sources are in Table S1.

Direct comparisons of measured variables within the mature forest to those of the plantations also are consistent with a belowground-feedback scenario. Although ANPP was greater in the plantations than in the mature forest, no significant differences in (H1) leaf area, leaf fall or total fine litterfall were observed between the sites, which also had similar leaf fall N contents (mg g^−1^) and fluxes based on the data available (Fig. 2, Table S1). Aboveground NPP (H2) averaged 22% higher in the plantations than in the mature forest, primarily due to 63% faster tree growth (Fig. 5). Aboveground biomass accumulated nearly six times faster in the plantations than in the mature forest (Fig. 5). Our estimate of BCA (H3) also was significantly greater in the plantations than in the mature forest (Fig. 2) despite the fact that we ignored probable differences in coarse-root biomass accumulation rates (Table S3), which likely mirrored the six-fold differences in net aboveground biomass accumulation. This finding is consistent with data from forests from different biomes summarized by [24], who found that forests <45 years old had relatively greater belowground C fluxes than did older forests. Overall, our findings are consistent with Fig. 1C in that both BCA and tree growth, but not canopy production, were significantly greater in the plantations than in the mature forest (Fig. 5).

Across a range of primarily mature tropical forests in Amazonia, ∼34% of estimated NPP was allocated to canopy production, with indications of a possible trade-off in allocation of production to woody stems versus fine roots [27], [60]. Such a tradeoff was inferred to be evolutionarily advantageous based on an individual-based forest growth model that was compared against empirical data [30]. Our results from mature forests and 15–20 year old plantations of native tree species in lowland Costa Rica were not consistent with such a trade-off: we found a significantly positive relationship between BCA and aboveground tree growth (Fig. 3, 4). We suggest that young forest plots that are accumulating biomass [10] and are sequestering nutrients from the soil [56] reflect disequilibrium conditions, and thus are likely to have different allocation patterns than do mature forests, if allocation patterns are flexible (*e.g*., Fig. 1B, 1C) and not fixed (Fig. 1A). Our data suggest flexibility in allocation of available growth substrates to canopies versus to root systems, with tree growth (wood production) in the plantations benefiting, in our case, from enhanced BCA. Clearly, healthy foliar canopies and root systems both contribute to ANPP: we presume that greater BCA in the experimental plantations at our site was related to soil degradation that occurred during pasture establishment and over the subsequent decades of low-level pasture management that preceded tree planting [40], [61]. This would be consistent with classical root-shoot allocation theory [12]. It also is generally consistent with a recent tree growth model [62] that posited that available C and N in trees were utilized to maximize annual wood production, with enhanced C fluxes to deep roots promoting N uptake in support of wood production. We infer that increasing allocation of C to root systems can be an important mechanism supporting high rates of tree growth and C sequestration in rapidly growing tropical forest plantations. Similar investigations of young forests at other sites are needed: identifying when, where and for how long enhanced allocation of C to root systems occurs during forest stand development would improve models of forest growth and forest-atmosphere C exchanges.

## Supporting Information

Table S1 **Attributes of mature forest and 15–20 year old tree plantations on residual soils at La Selva, Costa Rica.**
(DOCX)

Table S2 **Equations used to estimate per-tree aboveground biomass and leaf area in the plantations.**
(DOCX)

Table S3 **Belowground carbon fluxes in plantation and forest plots on residual soils at La Selva, Costa Rica.**
(DOCX)

Table S4 **Correlation matrix of carbon-flux variables used to investigate allocation patterns.**
(DOCX)
