# Correction: Aboveground Tree Growth Varies with Belowground Carbon Allocation in a Tropical Rainforest Environment

**DOI:** 10.1371/journal.pone.0117932

**Published:** 2015-02-10

**Authors:** The PLOS ONE Staff

The regressions equations in S2 Table for *V. guatemalensis* are incorrect. Please view the correct S2 Table below.

## Supporting Information

S2 Table Equations used to estimate per-tree aboveground biomass and leaf area in the plantations. Only trees ≥10 cm diameter at breast height were included in biomass and tree growth calculations for this study. For total aboveground biomass (TAB, kg) the independent variable was *d* ^2^ *h*, with *d* = tree diameter at breast height (m) and *h* = total tree height (m). For total tree leaf area (m^2^) the independent variable was *d* ^2^ (cm^2^). (DOCX)
