# Supplementary material for: Aboveground Tree Growth Varies with Belowground Carbon Allocation in a Tropical Rainforest Environment
Source: PLoS One. 2014 Jun 19;9(6):e100275. doi: 10.1371/journal.pone.0100275 (PMC4063787; doi:10.1371/journal.pone.0100275)
Supplement: Table S1 — Attributes of mature forest and 15–20 year old tree plantations on residual soils at La Selva, Costa Rica. (DOCX) [file pone.0100275.s001.docx]

**Table S1** Attributes of mature forest and 15-20 year old tree plantations on residual soils at La Selva, Costa Rica.

| **Plot Attribute (units)** | **Site** | **Interval** | **Mean** | ***n*** | ***95% CI*** | **Reference** |
| --- | --- | --- | --- | --- | --- | --- |
| Soil pH (0-10 cm) | F | 1998 | 4.2 | 12 | 4.1-4.3 | [1] |
| Soil pH (0-15 cm) | Pl | 2003, 2005 | 4.3 | 16 | 4.3-4.4 | [2] |
| SOC (0-30 cm) (Mg ha^-1^) | F | 1998 | 92 | 12 | 84-89 | [1] |
|  | Pl | 2003, 2005 | 86 | 16 | 83-90 | [2] |
| Soil N (0-30 cm) (Mg ha^-1^) | F | 1998 | 7.7 | 12 | 7.0-8.3 | [1] |
|  | Pl | 2003, 2005 | 6.8 | 16 | 6.6-7.0 | [2] |
| Soil C:N (0-30 cm) | F | 1998 | 12.0 | 12 | 11.8-12.2 | [1] |
|  | Pl | 2003, 2005 | 12.7 | 16 | 12.4-12.9 | [2] |
| Leaf fall (Mg ha^-1^ yr^-1^) | F | 2003-2009 | 7.1 | 12 | 6.5-7.7 | [3] |
|  | Pl | 2003-2009 | 7.5 | 16 | 7.2-7.8 | This study |
| Tree biomass (Mg ha^-1^) | F | 2003-2009 | 152 | 12 | 139-166 | [3] |
|  | Pl | 2003-2009 | 154 | 16 | 135-171 | This study |
| Tree growth (ABI, Mg ha^-1^ yr^-1^) | F | 2003-2009 | 4.6 | 12 | 4.3-4.9 | [3] |
|  | Pl | 2003-2009 | 7.5 | 16 | 6.8-8.2 | This study |
| Litterfall (Mg ha^-1^ yr^-1^) | F | 2003-2009 | 9.1 | 12 | 8.3-10.0 | [3] |
|  | Pl | 2003-2009 | 9.3 | 16 | 8.9-9.7 | This study |
| ANPP (Mg ha^-1^ yr^-1^) | F | 2003-2009 | 13.7 | 12 | 12.9-14.7 | [3] |
|  | Pl | 2003-2009 | 16.8 | 16 | 15.8-17.8 | This study |
| Leaf area index (m^2^ m^-2^) | F | 2003-2005 | 6.0 | 45 | 5.4-6.7 | [4] |
|  | Pl | 2003-2005 | 6.9 | 16 | 6.4-7.4 | This study |
| Δ(Forest biomass) (Mg ha^-1^ yr^-1^) | F | 2003-2009 | 1.1 | 12 | 0.1-2.1 | [3] |
|  | Pl | 2003-2009 | 6.4 | 16 | 5.3-7.4 | This study |
| Leaf fall [N] (mg g^-1^) | F | 1997-2001 | 16.6 | 12 | 16.1-17.3 | [5] |
|  | Pl | 2004-2005, 2009 | 17.2 | 16 | 16.7-17.8 | This study |
| Leaf fall N (kg ha^-1^ yr^-1^) | F | 1997-2001 | 120 | 12 | 110-132 | [5] |
|  | Pl | 2004-2005, 2009 | 128 | 16 | 122-134 | This study |
| R_SOIL_ (Mg C ha^-1^ yr^-1^) | F | 2005-2008 | 15.0 | 3 | 12.1-16.6 | This study |
|  | Pl | 2004-2010† | 18.5 | 16 | 17.6-19.5 | This study |
| BCA (R_SOIL_-LF) (Mg C ha^-1^ yr^-1^) | F | 2005-2008 | 10.3 | 3 | 8.3-12.3 | This study |
|  | Pl | 2004-2010 | 13.9 | 16 | 13.0-14.9 | This study |

Column headers: Site is either mature forest (F) or plantations (Pl); Interval refers to the years during which measurements were made; Mean is the observed average value based on the number of plots sampled (*n*); 95% *CI* is the confidence interval of the mean based on bootstrapping with 5000 draws.

Variables (column 1): Soil pH was measured in water; SOC is total soil organic carbon; Soil N is total soil N; Soil C:N is the mean plot-level ratio between SOC and Soil N; leaf area index is LAI; Leaf fall is all leaves collected in litterfall traps; Tree biomass is total aboveground biomass of all live trees ≥10 cm diameter; Tree growth is the total aboveground biomass increments of all trees ≥10 cm diameter; Litterfall is as measured from litterfall traps; ANPP is determined as Tree growth plus Litterfall; Δ(Forest biomass) is net aboveground forest biomass accumulation, *i.e*. tree growth minus tree mortality; Leaf fall [N] is the N content of leaf fall; Leaf fall N is the total N flux in leaf fall; R_SOIL_ is total soil respiration, or soil CO_2_ efflux; and BCA is belowground carbon allocation estimated from soil respiration minus litterfall.

References (column 7):

1. Espeleta JF, Clark DA (2007) Multi-scale variation in fine-root biomass in a tropical rain forest: A seven-year study. Ecol Monogr 77: 377-404.

2. Russell AE, Raich JW, Valverde-Barrantes OJ, Fisher RF (2007) Tree species effects on soil properties in experimental plantations in tropical moist forest. Soil Sci Soc Am J 71: 1389-1397.

3. Clark DA, Clark DB, Oberbauer SF (2013) Field-quantified responses of tropical rainforest aboveground productivity to increasing CO_2_ and climatic stress, 1997-2009. J Geophys Res Biogeosci 118: 783-794.

4. Clark DB, Olivas PC, Oberbauer SF, Clark DA, Ryan MG (2008) First direct landscape-scale measurement of tropical rain forest leaf area index, a key driver of global primary productivity. Ecol Lett 11: 163-172.

5. Wood TE, Lawrence D, Clark DA (2006) Determinants of leaf litter nutrient cycling in a tropical rain forest: Soil fertility versus topography. Ecosystems 9: 700-710.
