# Supplementary material for: Aboveground Tree Growth Varies with Belowground Carbon Allocation in a Tropical Rainforest Environment
Source: PLoS One. 2014 Jun 19;9(6):e100275. doi: 10.1371/journal.pone.0100275 (PMC4063787; doi:10.1371/journal.pone.0100275)
Supplement: Table S2 — Equations used to estimate per-tree aboveground biomass and leaf area in the plantations. (DOCX) [file pone.0100275.s002.docx]

**Table S2** Equations used to estimate per-tree aboveground biomass and leaf area in the plantations. Only trees ≥10 cm diameter at breast height were included in biomass and tree growth calculations for this study. For total aboveground biomass (TAB, kg) the independent variable was *d*^2^*h* (m^3^), with *d* = tree diameter at breast height (m) and *h* = total tree height (m). For total tree leaf area (m^2^) the independent variable was *d*^2^ (cm^2^).

| **Tree Species** | **variable** | **intercept** | **slope** | ***n*** | ***r*^2^** |
| --- | --- | --- | --- | --- | --- |
| *H. alchorneoides* | TAB | 48.00323 | 245.09583 | 9 | 0.99 |
| *H. alchorneoides* | LA | 19.66530 | 0.15666 | 9 | 0.86 |
| *P. macroloba* | TAB | 25.73019 | 207.71076 | 9 | 0.99 |
| *P. macroloba* | LA | 44.40218 | 63.62308 | 9 | 0.87 |
| *V. koschnyi* | TAB | 20.19554 | 159.62321 | 9 | 0.998 |
| *V. koschnyi* | LA | 24.46313 | 43.53941 | 9 | 0.96 |
| *V. guatemalensis* | TAB | -50.25019 | 146.74246 | 9 | 0.99 |
| *V. guatemalensis* | LA | -108.07841 | 0.14944 | 9 | 0.89 |
| Other trees (*d* ≥10 cm) | TAB | 23.31846 | 175.80698 | 54 | 0.91 |
| Other trees (*d* ≥10 cm) | LA | 3.31396 | 0.13847 | 54 | 0.85 |
| Saplings (*d* 2.5-10 cm) | TAB | -0.61191 | 0.20741 | 60 | 0.73 |
| Saplings (*d* 2.5-10 cm) | LA | 1.25807 | 0.24687 | 60 | 0.65 |
