# Supplementary material for: Aboveground Tree Growth Varies with Belowground Carbon Allocation in a Tropical Rainforest Environment
Source: PLoS One. 2014 Jun 19;9(6):e100275. doi: 10.1371/journal.pone.0100275 (PMC4063787; doi:10.1371/journal.pone.0100275)
Supplement: Table S3 — Belowground carbon fluxes in plantation and forest plots on residual soils at La Selva, Costa Rica. (DOCX) [file pone.0100275.s003.docx]

**Table S3** Belowground carbon fluxes in plantation and forest plots on residual soils at La Selva, Costa Rica. Data represent annual means in units of Mg C ha^-1^ year^-1^; the *SEM* is provided when it could be determined.

| **Variable** | **Mature Forest** | **Plantation** | **Reference** |
| --- | --- | --- | --- |
|  | **Mean (*SEM*)** | **Mean (*SEM*)** |  |
| R_SOIL_ | 15.0 (1.4) | 18.5 (0.70) | This study (Table S1) |
| Litterfall C | 4.6 (0.6) | 4.6 (0.2) | This study |
| Δ(Coarse-root biomass)* | 0.13† | 0.72† | estimated |
| BCA | 10.3 (1.2) | 13.9 (0.7) | = R_SOIL_ - Litterfall |
| Soil methane consumption | 0.003 | [0.003]§ | [1] |
| Litter | 2.8 (0.2) | 2.6 (0.2) | [2] |
| ΔLitter | negligible | negligible | [2] |
| ΔSOC† (accumulation rate) | 1.6 × 10^-4^ | 0.23 (0.04) | [3], [4] |
| DOC† in throughfall | 0.23 | no data | [5] |
| DIC† in throughfall | 0.0053‡ | no data | calculated‡ |
| DOC† leaching below 2.5 m | 0.058 (0.009) | no data | [5] |
| DIC† leaching below 2.5 m | 0.16 | no data | [6] |

* Estimated from (woody root:shoot) ×( net aboveground biomass increment) assuming a root:shoot of 0.235 [7].

† SOC is soil organic C; DOC is dissolved organic carbon; DIC is dissolved inorganic C; throughfall is rain that penetrates through the canopy to the forest floor including stemflow.

‡ Calculated using Henry's Law assuming throughfall of 3300 mm year^-1^ [8] and a soil-atmosphere CO_2_ concentration of 0.04% [6] at 25°C.

§ Secondary forests about 10 years older than the plantations but on the same soil type, for reference.

References (column 4)**:**

1. Keller M, Reiners WA (1994) Soil-atmosphere exchange of nitrous oxide, nitric oxide, and methane under secondary succession of pasture to forest in the Atlantic lowlands of Costa Rica. Global Biogeochem Cycles 8: 399-409.

2. Raich JW, Russell AE, Bedoya-Arrieta R (2007) Lignin and enhanced litter turnover in tree plantations of lowland Costa Rica. For Ecol Manage 239: 128-135.

3. Russell AE, Raich JW, Bedoya R, Valverde-Barrantes O, González E (2010) Impacts of individual tree species on carbon dynamics in a moist tropical forest environment. Ecol Appl 20: 1087–1100.

4. Veldkamp E, Becker A, Schwendenmann L, Clark DA, Schulte-Bisping H (2003) Substantial labile carbon stocks and microbial activity in deeply weathered soils below a tropical wet forest. Glob Chang Biol 9: 1171-1184.

5. Schwendenmann L, Veldkamp E (2005) The role of dissolved organic carbon, dissolved organic nitrogen, and dissolved inorganic nitrogen in a tropical wet forest ecosystem. Ecosystems 8: 339-351.

6. Schwendenmann L, Veldkamp E (2006) Long-term CO_2_ production from deeply weathered soils of a tropical rain forest: evidence for a potential positive feedback to climate warming. Glob Chang Biol 12: 1878-1893.

7. Mokany K, Raison RJ, Prokushkin AS (2006) Critical analysis of root:shoot ratios in terrestrial biomes. Glob Chang Biol 12: 84-96.

8. Loescher HW, Gholz HL, Jacobs JM, Oberbauer SF (2005) Energy dynamics and modeled evapotranspiration from a wet tropical forest in Costa Rica. J Hydrol (Amst) 315: 274-294.

**Table S4** Correlation matrix of C-flux variables related to carbon allocation patterns. Significant correlations are shown in underlined bold.

| Variable* | Leaf fall | Litterfall | Tree Growth | ANPP | BCA |
| --- | --- | --- | --- | --- | --- |
| Leaf fall | 1 | -- | -- | -- | -- |
| Litterfall | **0.88** | 1 | -- | -- | -- |
| Tree Growth | 0.14 | 0.25 | 1 | -- | -- |
| ANPP | **0.51** | **0.65** | **0.90** | 1 | -- |
| BCA | 0.19 | 0.11 | **0.53** | **0.48** | 1 |

* Tree growth is aboveground biomass increment of all trees ≥10 cm diameter; BCA is belowground carbon allocation.
