# Supplementary material for: Aboveground Tree Growth Varies with Belowground Carbon Allocation in a Tropical Rainforest Environment
Source: PLoS One. 2014 Jun 19;9(6):e100275. doi: 10.1371/journal.pone.0100275 (PMC4063787; doi:10.1371/journal.pone.0100275)
Supplement: Table S4 — Correlation matrix of carbon-flux variables used to investigate allocation patterns. (DOCX) [file pone.0100275.s004.docx]

**Table S4** Correlation matrix of C-flux variables related to carbon allocation patterns. Significant correlations are shown in underlined bold.

| Variable* | Leaf fall | Litterfall | Tree Growth | ANPP | BCA |
| --- | --- | --- | --- | --- | --- |
| Leaf fall | 1 | -- | -- | -- | -- |
| Litterfall | **0.88** | 1 | -- | -- | -- |
| Tree Growth | 0.14 | 0.25 | 1 | -- | -- |
| ANPP | **0.51** | **0.65** | **0.90** | 1 | -- |
| BCA | 0.19 | 0.11 | **0.53** | **0.48** | 1 |

* Tree growth is aboveground biomass increment of all trees ≥10 cm diameter; ANPP is aboveground net primary productivity; BCA is belowground carbon allocation.
